# Assessing the Solubility of Baricitinib and Drug Uptake in Different Tissues Using Absorption and Fluorescence Spectroscopies

**DOI:** 10.3390/pharmaceutics14122714

**Published:** 2022-12-04

**Authors:** Roya Mohammadi-Meyabadi, Negar Beirampour, Núria Garrós, Helen Lissette Alvarado, David Limón, Marcelle Silva-Abreu, Ana Cristina Calpena, Mireia Mallandrich

**Affiliations:** 1 Department of Pharmacy, Pharmaceutical Technology and Physical-Chemistry, Faculty of Pharmacy and Food Sciences, University of Barcelona, 08028 Barcelona, Spain; 2 Institut de Nanociència i Nanotecnologia IN2UB, Universitat de Barcelona, 08028 Barcelona, Spain; 3 Department of Pharmacology, Toxicology and Medicinal Chemistry, Faculty of Pharmacy and Food Sciences, University of Barcelona, 08028 Barcelona, Spain

**Keywords:** baricitinib, poorly water-soluble drug, solubility study, stability study, in vitro tissue uptake, fluorescence spectroscopy, absorption spectroscopy, drug recovery, drug uptake

## Abstract

The low water solubility of baricitinib (BCT) limits the development of new formulations for the topical delivery of the drug. The aims of this study were to assess the solubility of BCT in different solvents, including Transcutol, a biocompatible permeation enhancer that is miscible in water, to evaluate the drug uptake in human skin and porcine tissues (sclera, cornea, oral, sublingual, and vaginal), and to subsequently extract the drug from the tissues so as to determine the drug recovery using in vitro techniques. Analytical methods were developed and validated for the quantification of BCT in Transcutol using absorption and fluorescence spectroscopies, which are complementary to each other and permit the detection of the drug across a broad range of concentrations. Results show that Transcutol permits an increased drug solubility, and that BCT is able to penetrate the tissues studied. The solutions of BCT in Transcutol were stable for at least one week. Hence, Transcutol may be a suitable solvent for further development of topical formulations.

## 1. Introduction

Topical delivery is a noninvasive route and an alternative to oral administration. The topical route involves the skin and the nasal, buccal, sublingual, ophthalmic, rectal, and vaginal mucosae. Some patients struggle with oral administration, while in contrast the topical route is easy to administer, which may improve the patients’ compliance; some medications administered orally cause digestive side-effects, but the topical route may avoid this inconvenience. Additionally, drug abuse through the topical dosage form is lower than under oral administration. Topical delivery seeks the permeation of drugs through the skin or mucosae [1]. However, drugs face some barriers in penetrating into the tissues, these can include the presence of mucus on the mucosae, low water content, or the existence of the stratum corneum, which is the outermost skin layer and has the main barrier function [2].

**Figure 1:**
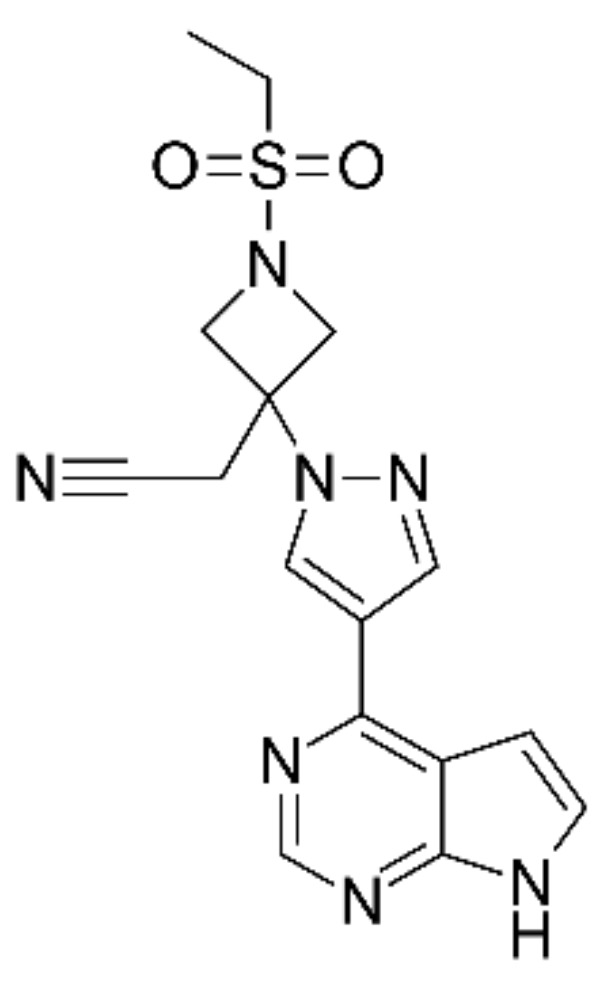
Chemical structure of BCT.

Baricitinib (BCT), named 2-[1-ethylsulfonyl-3-[4-(7 H-pyrrolo[2,3-d]pyrimidin-4-yl)pyrazol-1-yl]azetidin-3-yl]acetonitrile according to IUPAC (Figure 1), is an oral selective and reversible Janus-associated kinase (JAK) inhibitor, which modulates the signaling pathway. It has a known anti-inflammatory profile in patients with autoimmune diseases. The BCT mechanism of action consists of inhibiting the signal transduction of IL-6, IL-12, IL-20, IL-22, IL-23, and IFN-γ [3,4,5,6]. BCT was approved by the European Medicines Agency (EMA) for the treatment of moderately to severely active rheumatoid arthritis (RA) in adults and for the management of specific cases of atopic dermatitis [7]. Recently, the US Food and Drug Administration (FDA) approved BCT for emergency use in the treatment of COVID-19 due to its capability of modulating the immunopathology associated with SARS-CoV-2 infection, as well as for the treatment of alopecia areata in adults [8,9].

The therapeutic effectiveness and safety of BCT have been investigated, showing sufficient effectiveness and tolerability in clinical trials [10,11]. It is typically administered orally, after which 80% oral bioavailability has been reported in healthy human subjects, but it decreased by 11–18% in the presence of high-fat meals [8]. BCT has a relatively low molecular weight (371.42 Da), and, although BCT is very poorly soluble in water (0.357 mg/mL to 0.46 mg/mL at 25 °C) [3,12], it is bound to plasma and serum proteins; thus, its oral administration implies systemic distribution. After intravenous administration, its volume of distribution is high (76 L), confirming that distribution to the tissues is significant, which is also in line with its low water solubility.

The typical oral doses range from 1 to 4 mg, indicating that BCT is a very potent drug, especially when its high distribution volume is considered [13]. Therefore, oral administration requires sufficiently high doses so as to achieve therapeutic concentrations in the tissues undergoing inflammatory processes. However, at the same time, it implies potential secondary effects.

The achievement of efficacy while decreasing potential secondary effects can be achieved by topical administration, using administration routes such as dermal, ophthalmic, injectable, etc. [6,14,15].

Formulation approaches for the enhancement of the bioavailability of BCT are very scarce in the literature [16,17], most probably due to its very low solubility in water. Moreover, BCT is poorly soluble in ethanol (0.40 mg/mL), but it is freely soluble in organic solvents such as dimethyl sulfoxide (74 to 165.1 mg/mL) and dimethylformamide (50 mg/mL) [12,16]. The toxicity of the organic solvents might have also influenced in the lack of suitable formulations for alternative routes of administration. In contrast, BCT is soluble in PEG-400 (72.4 mg/mL) [12], which is in turn soluble in water. However, PEG-400 is a polymer of nine units of ethylene glycol, and, although it is considered biocompatible, it has been reported that increasing the number of ethylene glycol units of the PEG decreases intestinal permeability [18]. Apart from these solvents, the solubility of BCT in other solvents or its stability in solution have not been studied systematically. Moreover, some analytical methods have been developed to quantify BCT, such as high-performance liquid chromatography (HPLC) and liquid chromatography–mass spectrometry (LCMS/MS). However, despite their advantages such as repeatability, high sensitivity, and reliability, HPLC methods imply a high cost, complex data processing, and greater time consumption. In contrast, UV/Vis absorption spectroscopy or fluorescence spectroscopy which has advantages over other methods, such as its ease and simplicity, fair sensitivity, relatively low cost, and low time consumption [19,20,21]. Although few analytical methods using these techniques have been developed for BCT [22], the absorption range of absorption wavelengths of BCT in the UV region implies high interference from other compounds, especially biological components, for which it limits the quantification of BCT from biological samples. Furthermore, fluorescence spectroscopy on the other hand is a suitable and simple, inexpensive, rapid, and reproducible technique used for evaluating fluorescent compounds [23]. Compared to absorption spectroscopy, fluorescence spectroscopy is much more sensitive, permitting much lower limits of detection (LOD) and limits of quantification (LOQ). Because of the low solubility of BCT, the assessment of its in vitro availability in different tissues upon topical administration might require highly sensitive techniques such as this one.

Transcutol, also called diethylene glycol monoethyl ether, is a polymer of three units of ethylene glycol, and it is known to be a biocompatible solvent and permeation enhancer [24]. It is a clear and colorless liquid, which is water-soluble with a melting point of −76 °C [25]. It is widely used in pharmaceutical products, cosmetics, and food because of its low toxicity and high capacity as a solubilizer. Transcutol has been used in oral and sublingual solutions, as well as in injectable products. It has been included in creams, emulsions, gels, ointments, and solutions for topical delivery, covering a broad range of drugs and applications, including analgesic, anti-inflammatory, antifungal, hormones, and veterinary products [26]. Despite these advantages, Transcutol has never been used as a solvent for BCT in any studies. We should take into account that baricitinib presents severe side-effects, and the topical route is an alternative to the oral one, especially when local effects are intended. For instance, patients with atopic dermatitis who do not require systemic immunosuppressant therapy might benefit from topical formulations because this route may avoid systemic side-effects.

The aims of this study were firstly (i) to test the solubility of BCT in different solvents (aqueous solutions, oils, surfactants, and permeation enhancers such as Transcutol). It was to be a preliminary and pre-formulation study before leading to further developing formulations for topical delivery; (ii) to evaluate the uptake of the drug in different tissues (buccal, sublingual, nasal, vaginal, corneal, scleral mucosae, and skin) and the drug recovery; and (iii) to validate the absorption and fluorescence spectroscopy methods. As part of the validation, the stability of BCT solutions in Transcutol was studied at different temperatures. 

## 2. Materials and Methods

### 2.1. Chemicals and Reagents

The BCT bulk powder ingredient was supplied by Henrikang Biotech Co., Ltd. (Xi’an, China). Transcutol, Labrafac, Isostearyl Isostearate, Labrasol, Lauroglycol 90, Labrafil M 1944 CS, Plurol oleique, and Capryol 90 were acquired from Gattefossé (Saint-Priest, France). Dimethyl sulfoxide (DMSO), Tween-80, and oleic acid were provided by Panreac Química SA (Barcelona, Spain); limonene, α-pinene, nonane, 1-decanol 99%, octanoic acid, lauryl sulfate, sebacic acid, castor oil, and phosphate-buffered solution pH 7.4 were purchased from Sigma Aldrich (St. Louis, MO, USA); Surfadone LP 100, N-ethyl pyrrolidone (NEP), and *N*-methyl pyrrolidone were obtained from ISP (West Yorkshire, UK). Perhydro Squalene was acquired from Fagron Iberica (Terrassa, Spain), liquid paraffin was supplied by Roig Farma, (Terrassa, Spain), and distilled water and purified water were obtained using a Station 9000 purification unit.

### 2.2. Biological Material

In this work, different tissues were used to assess the drug uptake: buccal, sublingual, vaginal, corneal, and scleral mucosae were of porcine origin, and ex vivo human skin was employed. Porcine specimens were obtained under veterinary supervision from residual individuals of female pigs (cross Landrace × Large White, 25–30 kg) in accordance with the protocol described by Pérez-González and coworkers [27]. In accordance with the 3R rules, the animals had been used previously in surgical university practices, in accordance with the Ethics Committee of Animals Experimentation at the University of Barcelona. The tissues were immediately transported to the laboratory immersed in artificial aqueous humor solution to be debrided and plain-prepared for the experiments. Human skin was obtained from abdominoplasties practiced on healthy women (Barcelona SCIAS Hospital, Barcelona, Spain). The Bioethics Committee of the Barcelona-SCIAS Hospital approved the study protocol (N°002; dated 17 January 2020). The skin was stored at −20 °C until the experiments were carried out.

### 2.3. Screening of Solubility in Different Oils and Enhancers

Solubility is a common challenge faced in the development of galenic formulations, as many drugs are poorly water-soluble. Actually, about 40% of new chemical entities are water-insoluble. Taking into account that only a fraction of the drug dissolved will be absorbed at the absorption site, several strategies have been used to enhance the solubility of poorly water-soluble drugs. Before going into any pharmaceutical development, the solubility of BCT was assessed in different solvents, including aqueous media, oils, and other kinds of solutions.

The solubility study was evaluated by the subsequent addition of BCT to 10 mL of solvent, using continuous sonication of each addition at 25 °C for 20 min. Small amounts of BCT (tip of a spatula) were added to the solvent until saturation was observed. The solutions were filtered through a 0.45 μm pore size nylon membrane and analyzed using absorption spectroscopy or fluorescence spectroscopy (see Section 2.5). The following solvents were tested: Transcutol, Labrafac, Isostearyl Isostearate, Lauroglycol 90, Capryol 90, Limonene 97%, α-pinene, 1-decanol 99%, lauryl sulfate, sebacic acid, castor oil, Surfadone LP 100, *N*-ethyl pyrrolidone, N-methyl pyrrolidone, liquid paraffin, distilled water, phosphate-buffered solution pH 7.4 (PBS), and mixtures of PBS:Transcutol. Of all the solvents tested, the selection of the solvent for the drug recovery assay (Section 2.4) was based on the solubilization capacity of the solvent also taking into account the biocompatibility of the solvent with the tissues.

### 2.4. Drug uptake and Drug Recovery from the Skin and Mucosae

In topical delivery, the target may be local or systemic. When the local effect is intended, the target sites may be the tissues beneath the site of application or deeper regions [28]. Thus, it is important to evaluate the capacity of the tissues of interest to uptake a given drug. For this purpose, the recovery of BCT in different tissues was performed. The following tissues were included in this study: human skin, and porcine buccal, sublingual, vaginal, corneal, and scleral mucosae. The skin and some mucosae were cut in horizontal sections using a dermatome (GA630, Aesculap, Tuttlingen, Germany) at 0.4 mm thickness for the skin and at 0.5 mm for the following mucosae: nasal, buccal, and sublingual. This thickness is commonly used in ex vivo permeation assays [29,30]. In the case of the skin, it presents its main representative layers (stratum corneum, viable epidermis, and part of the dermis). The cornea and sclera were isolated from the eyeball and used at full thickness [31], and the same process was followed for the vaginal mucosa [32].

**Figure 2:**
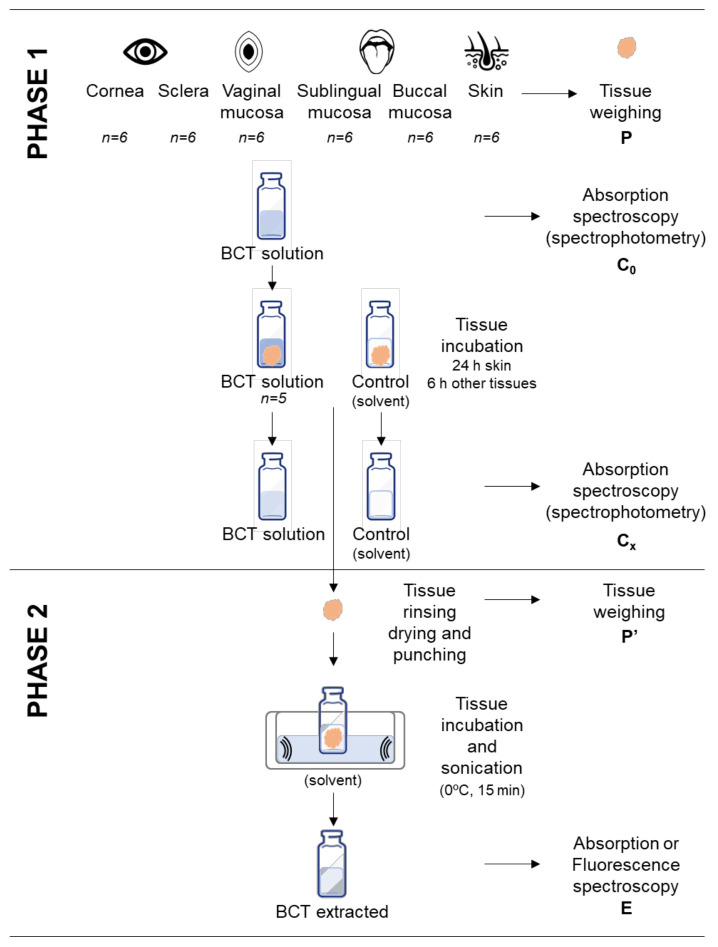
Schematic representation of the recovery process comprising phase 1 (drug uptake) and phase 2 (drug extraction). Samples consisted of pieces of tissue incubated in a solution of baricitinib, and the controls were tissues incubated in the solvent without baricitinib so as to evaluate any interference from the tissue with the analytical methods.

The goal of this assay is to assess the amount of drug able to penetrate the tissue (uptake) and the efficacy of the extraction method (recovery). Stated succinctly, recovery consists of two phases: the uptake process (phase 1), followed by the drug extraction process (phase 2), as summarized in Figure 2.

To perform phase 1, a plain solution of BCT in Transcutol was prepared at a known concentration (C_0_). Next, 4 mL of this solution were added to pieces of tissues of 0.64 cm^2^, which had been previously weighed. The tissues included in the study were previously evaluated for integrity by means of the measurement of transepidermal water loss (TEWL) or transmucosal water loss (TMWL), and only those tissues that met the criterium were used [33,34,35]. The vials were placed in a water bath at 32 °C for cornea and skin, and at 37 °C for the remaining tissues, corresponding to the cutaneous and body temperatures, respectively. The incubation time was 24 h for the skin and 6 h for the other tissues. These periods are common in the duration of the ex vivo permeation assays [29,30,32,36,37]. The C_0_ solution was also incubated in the water bath in the same conditions as the tissues so as to evaluate any potential degradation of the drug in the experiment conditions. Control samples of each tissue were also incubated in Transcutol without BCT in the same conditions. The study was conducted in sextuplicate. After the incubation process, the supernatants were collected for further analysis. Absorption spectroscopy (spectrophotometric method) was used to analyze the concentrations of the solutions before incubation (C_0_) and after incubation (C_x_) solutions [38]. The difference in concentration before and after incubation corresponded to the drug uptake by the tissue, while taking into account the control tissues with Transcutol (the solvent) without the drug. This permitted the evaluation of any interference of the tissue components during the sample analysis [39,40].

After the incubation for the drug uptake, the tissues were rinsed with purified water, blotted, punched, and weighed again for the subsequent drug extraction (phase 2). Then, 1 mL of Transcutol was used as the extraction solvent, which was added to the vials containing the drug-loaded tissues, and then the vials were sonicated in an ultrasonic water bath for 15 min in ice to prevent thermal degradation. The supernatant of each sample was analyzed by a fluorimeter or spectrophotometer depending on the sample concentration yielding the drug extracted from the tissues. Drug recovery is the relationship between the drug that has penetrated the tissue (uptake) and the drug extracted from the tissue, and it is expressed as a percentage. The percentage recovery (R%) was calculated using Equation (1):

(1) $$R\%=\frac{\frac{E}{P^{\prime }}}{\frac{C_{0}-C_{x}}{P}}\times 100,$$

where *E* is the concentration of BCT after extraction process (phase 2), *P*′ is the weight of tissue after extraction, *P* is the weight of tissue in the uptake phase (phase 1), *C*_0_ is the initial solution at a known concentration of BCT, and *C*_*x*_ is the concentration of the supernatant after the incubation (uptake phase, phase 1).

### 2.5. Analytical Methods

The wide concentration range of BCT shown in solubility studies and in recovery studies requires the development of two analytical methods which are complimentary to each other, and for which absorption spectroscopy (spectrophotometric method) and fluorescence spectroscopy (fluorometric method) were used. As fluorescence spectroscopy is much more sensitive than absorption spectroscopy, it permits the analysis of BCT concentration in solvents in which it is poorly soluble or in samples from recovery experiments (once the drug has been incorporated and extracted back from the tissue), whereas absorption spectroscopy permits the analysis of higher concentrations of BCT, such as those handled during the uptake into the tissues (see Section 2.4). The solvent used to develop and validate the analytical methods was based on high solubility and tolerability.

#### 2.5.1. Preparation of the Standard Stock Solution

The standard stock solution was prepared by dissolving 20 mg of drug in 20 mL of Transcutol to get the concentration of 1000 µg/mL.

#### 2.5.2. Preparation of the Calibration Curve

All calibration curve solutions were prepared using Transcutol as the solvent. Starting from the stock solution (1000 µg/mL), dilutions were prepared in the following concentrations for absorption spectroscopy: 60, 50, 40, 30, 25, 20, 15, 10, 7.5, and 6.25 μg/mL, whereas the following concentrations were established for fluorescence spectroscopy: 1.25, 0.625, 0.312, 0.156, and 0.078 μg/mL. 

#### 2.5.3. Absorption Spectroscopy (Spectrophotometric Method)

Absorbance measurements were performed using a PerkinElmer UV/Vis spectrophotometer (Shelton, CT, USA) and a 1 cm quartz cuvette. First, a spectrum was acquired in the wavelength range 250 nm to 400 nm. The maximum absorption was observed at 310 nm, for which absorbance values were read at this wavelength. A control experiment was carried out simultaneously in Transcutol (without BCT).

#### 2.5.4. Fluorescence Spectroscopy (Fluorometric Method)

The excitation and emission spectra were obtained using a RF-1501 Fluorimeter (Shimadzu, Canada) (light source = 150 W xenon lamp in self-contained lamp housing), managed by FL Solutions software and using a quartz cuvette. On the basis of the results, the fluorometric conditions to quantify the concentrations of BCT on all samples were an excitation wavelength of 310 nm and an emission wavelength of 390 nm. A control experiment was carried out simultaneously in Transcutol (without BCT).

### 2.6. Validation of the Analytical Methods

Validation concerned the evaluation of linearity, range, accuracy, and precision in accordance with the standard rules of the European Medicines Agency [41] and *Asociación Española de Farmacéuticos de la Industria* [42]. The limits of detection and quantification of the methods were also determined. The stability of the standard solutions was also evaluated.

#### 2.6.1. Linearity and Range

Linearity is the ability within a defined range to obtain results directly proportional to the concentrations of the analyte in the sample [41]. The range is the interval defined by the upper and lower concentrations of the tested drug for which it has been proven that the method has a suitable level of accuracy, precision, and linearity [41]. Linearity was assessed with five calibration curves at 10 concentration levels for the spectrophotometric method and five concentration levels for the fluorometric method. Individual slopes between the instrumental signals versus the corresponding drug concentrations were calculated. The least-squares regression was calculated using Equation (2), reporting the corresponding determination coefficients (r^2^).

(2) Abs=S·C+a,

where *C* is the concentration, *Abs* is the absorbance in the spectrophotometric method or the emission in the fluorometric method, *S* is the value of the slope, and *a* is the y-intercept. One-way analysis of variance (ANOVA) was performed to compare the signals versus nominal concentration at each concentration searching for nonsignificant differences (significance level set at *p* < 0.05). Results were processed using the Prism^®^, V.5.00 software (GraphPad Software Inc., San Diego, CA, USA).

#### 2.6.2. Sensitivity

The limit of detection (LOD) is the lowest amount of analyte in the sample that can be detected by the method, and the limit of quantification (LOQ) is the lowest amount of analyte in the sample that can be determined by precision, accuracy, and linearity. The LOD and LOQ were calculated according to the standard deviation of the response and the slope of the calibration curve using Equation (3):

(3) $$LOD\;or\;LOQ=\frac{K\times SD_{a}}{S},$$

where *K* is a factor related to the level of confidence (3.3 for *LOD* and 10 for *LOQ*). *SDa* is the standard deviation of the intercept (*a*), and *S* is the slope of the calibration line.

#### 2.6.3. Accuracy and Precision

The accuracy of an analytical method indicates the capacity of the method to provide results close to the real value. In this study, the accuracy was evaluated using standard solutions of five calibration curves: within the concentration range of 6.25 to 60 μg/mL for spectrophotometry and within 0.078 to 1.25 μg/mL for fluorometry. The accuracy at each concentration level was expressed as the mean percentage deviation or relative error (RE%) with respect to the nominal concentration, calculated using Equation (4).

(4) $$\%RE=\frac{C_{obs}-C_{nom}}{C_{nom}}\times 100,$$

where *C_obs_* is the observed concentration, and *C_nom_* is the nominal concentration of each standard solution.

Precision is the degree of variance between measurements. To assess the precision of the method, the standard solutions were prepared by two different analysts on two different days (intermediate precision), and it was expressed as the relative standard deviation (RSD%) of the different replicates at the different concentration levels, using Equation (5).

(5) $$\%RSD=\frac{SD}{C_{nom}}\times 100,$$

where *SD* is the standard deviation of the replicates at a certain concentration, and *C_nom_* is the nominal concentration.

#### 2.6.4. Stability

The stability of standard solutions was evaluated at two levels, the lowest and the highest concentration of the calibration curves of both methods, i.e., 6.25 µg/mL and 60 µg/mL for the spectrophotometric method, and 0.078 µg/mL and 1.25 µg/mL for the fluorometric method. The four solutions were incubated at three different temperatures: room (22 ± 2 °C), refrigerator (5 ± 3 °C), and freezer (−20 ± 5 °C).

The standard solutions were analyzed at time 0 (freshly prepared), on day 1, and on day 7. The stability was calculated as the relative difference between the absorbances at each stability point and the absorbance of the freshly prepared solution, as shown in Equation (6).

(6) $$Relative\;difference\;\left(\%\right)=\frac{Abs_{t}-Abs_{t0}}{Abs_{t0}}\times 100,$$

where *Abs_t_* is the absorbance of the standard solution at the stability point, and *Abs_t*0*_* is the absorbance of the standard solution just prepared.

## 3. Results and Discussion

### 3.1. Solubility Studies in Different Oils and Enhancers

Solubility is one of the most important physicochemical attributes for drug development, as low solubility can hinder the development of different products and severely limit the bioavailability of orally administered dosage forms [43]. Moreover, low aqueous solubility (<1 mg/mL) [44] is the main problem encountered in the development of formulations of new chemical entities [45,46]. The estimation of the solubility using the solid dispersion method for a given drug in different solvents and cosolvents is important for the development of future formulations which can increase the bioavailability of the drug of interest [12,47,48]. In this study, the solubility of BCT was evaluated in the aqueous solvents reported in Table 1. To this end, the saturated solutions prepared in the different solvents (see Section 2.3) were filtered and analyzed by absorption spectroscopy or fluorescence spectroscopy using the validated methods. According to the range of concentrations in which the analytical methods were developed and validated, Table 1 and Table 2 show the solubilities estimated using absorption spectroscopy, whereas Table 3 shows those estimated using fluorescence spectroscopy.

The solubility of BCT was tested in pure water, phosphate-buffered saline at pH 7.4, and mixtures of both solvents. Despite being a poorly water-soluble drug (<1 mg/mL) [44], the European Medicines Agency classifies it as Class III in the Biopharmaceutical Classification System (BCS) [49], i.e., poorly permeable and highly soluble [16]. This discrepancy in the classification of baricitinib as poorly or highly water-soluble is due to the different criteria used to classify the drug’s solubility. While the USP indicates that a drug is poorly soluble when its solubility is below 1 mg/mL [44], the grade of solubility in the BCS is defined as highly soluble when “the highest dose strength is soluble in less than 250 mL water over a pH range of 1 to 7.5” [50]. Considering that the highest strength of baricitinib is 4 mg, the resulting concentration in 250 mL of water is much lower than 1 mg/mL (i.e., soluble). However, according to the BCS criteria, the drug is highly soluble. The addition of Transcutol to PBS increases the amount of BCT that the medium is able to dissolve. A low proportion of Transcutol slightly improved the solubility profile of BCT in PBS, and better results were observed when the proportion of Transcutol is 50%, which increased the solubility of BCT more than 100 fold.

The greatest solubility was found to be in N-ethyl pyrrolidone, followed by Transcutol (Table 2).

An effective strategy to enhance drug permeation is to increase the concentration of drug dissolved because the rate of permeation (flux) increases proportionally to the drug concentration due to an increase in the thermodynamic activity together with the partition of the drug [1,51]. Bolla and coworkers investigated the solubility of ibuprofen in different solvents including Transcutol, propylene glycol, and isopropyl myristate. The greatest solubility was observed in Transcutol and propylene glycol. The researchers evaluated the permeability in vitro of ibuprofen formulated in different dosage forms (creams, hydrogels, and nonaqueous gels), and they observed that the nonaqueous gels containing Transcutol and propylene glycol exhibited higher amounts of drug permeated per unit area [51]. Hence, the solvent for developing topical formulations must be selected very carefully as it may have a great impact on drug permeability.

Some compounds are able to promote the rate and extent of drugs permeated into the tissues by disrupting the barrier function of the tissues. These are defined as penetration enhancers and are added to the formulations to increase or modulate the permeation of drugs. The penetration enhancers should be nontoxic, nonirritant, and non-sensitizing, with nonpharmacological activity, and they should exhibit a rapid onset of action in reducing the tissue barrier resistance while maintaining a reversible effect, whereby the skin or mucosa can recover their properties. Moreover, penetration enhancers should also be compatible with the formulation.

Transcutol is considered nontoxic and biocompatible with the skin and other tissues. It is listed in the Food and Drug Administration in the United States at 5% for transdermal use and at 25% for topical use. Health Canada approved Transcutol as a natural health product, and in 2002, the Cosmetic Ingredient Review expert panel evaluated 622 products containing Transcutol from 0.0004% to 80% and concluded that Transcutol is safe for use in cosmetics [26]. Gad et al. investigated the toxicity of Transcutol in different animal models and routes of administration; for instance, Transcutol was well tolerated in cats when administered intravenously at 2 mL/kg. The researchers also evaluated the tolerability of 50% Transcutol applied dermally in rabbits on a skin surface area of 2 cm^2^ with no signs of skin irritation. The acute toxicity study was conducted in rats administering 5 g/kg orally; the lethal dose 50 (LD 50) was established above 5000 mg/kg [52]. Considering Transcutol’s capacity in solubilizing BCT and its safety profile, it was selected as the vehicle for the subsequent study of drug uptake by different tissues and drug recovery from the tissues (Section 3.2).

### 3.2. Drug Uptake by the Tissues and Recovery from the Skin and Mucosae

The amount of BCT available in different ex vivo tissues was studied by carrying out recovery experiments. Firstly, the tissues were incubated in a solution of BCT in Transcutol at a known concentration for 6 or 24 h at 32 or 37 °C (drug uptake phase—phase 1), where the amount of BCT that can penetrate and be retained within different tissues, and thus the amount of BCT that can be extracted from the tissues (recovery phase—phase 2) were determined. This methodology is commonly used in dermal and transdermal delivery to determine the percentage recovery of the drug using a specific extraction method. The recovery allows the calculation of the amount of drug retained in the skin from the extraction data by multiplying the result by the recovery factor. Silva-Abreu and coworkers studied the permeation of pioglitazone loaded in polymeric nanoparticles in ex vivo mucosae, including buccal, sublingual, nasal, and intestinal from porcine origin. After the permeation study, the researchers assessed the amount of pioglitazone uptake by the mucosae by extracting the drug with a solvent under an ultrasonic bath treatment and subsequent correction by the recovery factor [53]. Gómez-Segura et al. used the same technique to determine the amount of carprofen uptake by ex vivo conjunctiva, cornea, sclera, buccal, sublingual, and vaginal mucosae [54]. This technique has also been applied to in vivo studies; Miralles et al. introduced pioglitazone nanoparticles into the eyes of pigs and analyzed the samples from the aqueous humor, vitreous humor, cornea, sclera, and lens after a drug extraction procedure. The study revealed that the drug was mainly located in the sclera [55].

In this work, results show that similar amounts of BCT per gram of tissue could penetrate the different tissues studied, except for the buccal mucosa, which could incorporate the greatest amount of BCT (208.68 µg/g). From the BCT incorporated within the tissue, the extraction procedure was able to recover 27.52% for the cornea, 25.01% for the sublingual, and 16.44% for buccal mucosae. Recoveries below 10% were found for the remaining tissues (Table 4).

The differences in the drug amount that penetrates the tissues may be due to structural differences between the tissues. The skin and the cornea are stratified, and each layer differs in water, lipid, and protein content [12]. The skin is the largest organ of the body; it represents about 15% of the body weight with a surface area of about 2 m^2^ [1] and an average thickness of 2–3 mm depending on the age and anatomical site. Additionally, the skin’s outermost layer (the stratum corneum) is a keratinized structure, which is the main barrier to drug permeation [56] with pH ranging 4.5–6 [57]. The low penetration in the skin may be due to the stratum corneum which hinders drug permeation. The oral cavity has a surface area of about 200 cm^2^, of which 50 cm^2^ correspond to the buccal mucosa and 25 cm^2^ to the sublingual mucosa. The nasal mucosa covers about 130 cm^2^. The pH of these mucosae ranges from 5.0–7.5 approximately, with a thickness of 0.7–1 mm for the nasal, 0.5–0.8 mm for the buccal, and 0.1–0.2 mm for the sublingual mucosae. Concerning the water content, the fluid volume on all three mucosae was 0.1–1 mL [2]. The administration of drugs via the vaginal route has been extensively used, either for local therapy to manage different infections and spermicidal agents, or for systemic therapy such as hormone supply [58]. The vaginal epithelium is stratified and non-keratinized with a thickness of 0.2–0.5 mm. The vaginal fluid (including the vaginal mucus) covers the epithelium to protect the vagina against pathogen infections, while acting as a barrier to drug penetration [59].

Ocular drug delivery faces physiological and anatomical barriers, tears with a volume of about 7 µL clear more than 95% of the applied dose in less than half a minute after instillation. The cornea is a transparent tissue of about 500 µm thickness [60] and offers significant resistance to drug permeation because of the tight junctions between epithelial cells, in addition to its lipidic nature. The sclera is composed of collagen fibers and proteoglycans with a thickness of about 300 µm [60]. The permeability of the drugs is affected by the size and charge of the molecules, being inversely proportional to the size and resulting in lower permeability of those molecules with positive charge [61].

The mucus covers the mucosal surface including the gastrointestinal tract, vagina, and eyes. In addition to lubricating and hydrating the epithelia, it acts as a barrier to drug penetration. It is mainly composed of mucins and water. The thickness of the mucus differs in the different mucosae; it is approximately 10 µm for the nasal mucosa, 85 µm for the buccal and sublingual mucosae, 50 µm for the vaginal mucosa, and between 3 to 30 µm on the eye [2,62,63].

The characteristics and particularities of the tissues and the existing barriers limit the bioavailability of the drug; hence, strategies to increase drug solubility and to overcome the skin and mucosal barriers are often required in topical delivery, and which include the use of physical or chemical enhancers, micro and nanoemulsions, nanoparticles, nanocrystals, lipid-based formulations, micelle-based formulations, etc. [64].

Another point to take into account is that the permeation of a given drug depends on the drug’s physicochemical properties, as well as on the partition coefficient formulation/tissue, which is indicative of the affinity of the drug for the vehicle in which it is formulated and for its affinity to the tissue. It is possible to elucidate the mechanism involved in the permeation of the drug by calculating the diffusion and partition coefficients after ex vivo permeation tests. Mallandrich and coworkers evaluated the permeation of ketorolac loaded in polymeric nanoparticles through corneal and scleral tissues. The researchers found that the diffusion coefficient had similar values in the cornea and sclera, and the effect of the partition coefficient was greater than that of the diffusion coefficient, and this parameter differed between the cornea and the sclera, indicating that the tissue has an influence on the permeation of the drug [32]. In this study, the uptake and recovery studies were conducted with the same drug in the same vehicle but in different tissues. Then, further investigation by the ex vivo permeation test using the tissues involved in this study allowed the determining of the partition and diffusion coefficients and shed light on the impact of the tissue on drug permeation [1].

Another aspect to consider is that the tissue pieces were immersed in the solution of baricitinib, and the drug probably penetrated the tissues through the surface, as well as through the inner part of the tissue and via a lateral entrance. In an in vivo application, only the surface of tissue is in contact with the solution; since the permeation of the drug is surface-dependent, the drug uptake may be higher under these conditions than in real applications. Nevertheless, the goal of the recovery study was not to evaluate the extent of permeation itself, but to determine the efficacy of the extraction method so as to obtain the correction factor for calculating the drug remaining in the skin after ex vivo permeation tests, thus informing of the rate and extent of permeation for each tissue.

### 3.3. Analytical Methods Validation

So as to develop appropriate delivery systems for topical routes of administration, adequate solvents must be first found. They must be biocompatible and they must permit a sufficient amount of dissolved drug. Then, analytical methods must be developed and validated for the quantification of the drug in different samples.

Due to its low water solubility, BCT was added and dissolved in different oils and solutions (10 mL) using continuous sonication until saturation was observed. The following solvents were tested: Transcutol, Labrafac, Isostearyl Isostearate, Lauroglycol 90, Capryol 90, limonene 97%, α-pinene, 1-decanol 99%, lauryl sulfate, sebacic acid, and castor oil, Surfadone LP 100, *N*-ethyl pyrrolidone, *N*-methyl pyrrolidone, and liquid paraffin.

Of the solvents tested, *N*-ethyl pyrrolidone was the solvent which showed the highest amount of BCT dissolved, followed by Transcutol. However, *N*-methyl pyrrolidone shows high cytotoxicity, whereas Transcutol is highly biocompatible [65,66]. For this reason, Transcutol was selected for further recovery experiments in different tissues and, consequently, also used as the solvent to develop the analytical methods.

**Figure 3:**
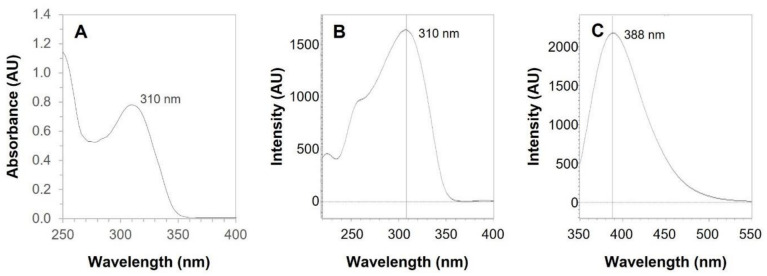
(**A**) Absorption spectrum of BCT in Transcutol. (**B**) Excitation spectrum of BCT in Transcutol. (**C**) Emission spectrum of BCT in Transcutol.

Absorption spectroscopy of BCT in Transcutol shows a strong absorption at 310 nm (UV range) corresponding to BCT (Figure 3A), which is in accordance with the extended pi conjugation in the molecule (Figure 1).

Similarly, fluorescence spectroscopy showed a maximum excitation at 310 nm (Figure 3B) and a maximum emission at 388 nm (Figure 3C). 

### 3.4. Validation of the Analytical Methods

Calibration curves were prepared from a stock solution (1000 µg/mL) of BCT in Transcutol, from which dilutions were also prepared using Transcutol and analyzed using absorption spectroscopy or fluorescence spectroscopy, in both cases using excitation at λ 310 nm.

For each technique used, five replicates were analyzed, in accordance with the validation guidelines by European Medicines Agency Guideline and Asociación Española de Farmacéuticos de la Industria which indicated that between three to six replicates for each validation should be included [41,42].

#### 3.4.1. Linearity and Range

**Figure 4:**
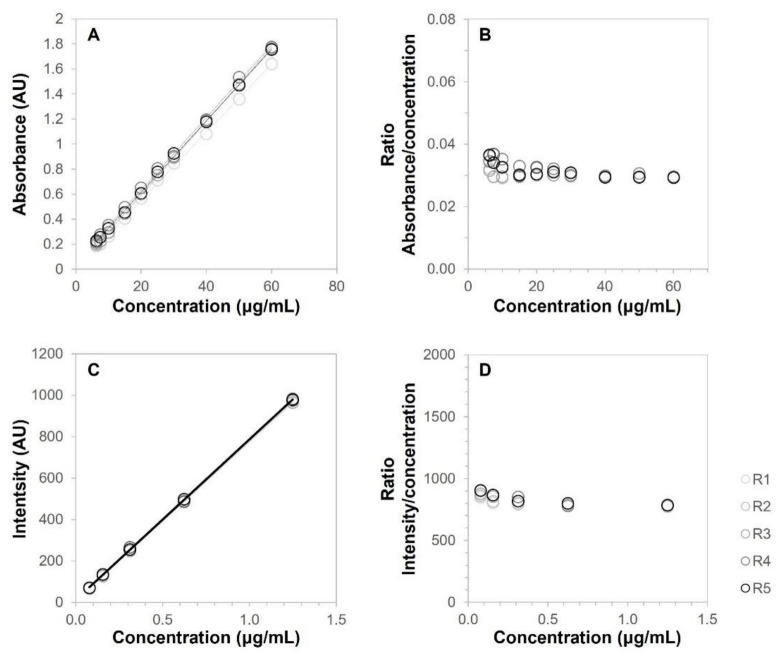
(**A**) Calibration curves of BCT in Transcutol (*n* = 5) using absorption spectroscopy. (**B**) Response factor (ratio absorbance/concentration) using absorption spectroscopy. (**C**) Calibration curves of BCT in Transcutol (*n* = 5) using fluorescence spectroscopy. (**D**) Response factor (ratio intensity/concentration) using fluorescence spectroscopy.

Figure 4A shows the calibration curves of BCT in Transcutol using absorption spectroscopy. The linearity of the calibration curves was assessed according to the correlation coefficient. All values of r^2^ were above 0.9994. Furthermore, Figure 4B shows a constant response factor (ratio absorbance/concentration) in the concentration range of 6.52 μg/mL to 60 μg/mL, with only slighter variation at the lowest concentrations. Table 5 shows the response factors obtained using absorption spectroscopy, whereas Table 6 shows the response factors using fluorescence spectroscopy. No statistical differences (statistical significance set at *p* < 0.05) were found after ANOVA analysis of the response factors using either technique.

The response factor is defined as the ratio between the response of the detector (signal) and the concentration of the analyte. This parameter correlates the signal to the concentration. When a method is linear, the signal increases proportionally to the analyte concentration; thus, the response factor remains constant for the different concentration levels. An ANOVA test of the response factor showed that there were no statistical differences in response factors over the analyte concentrations and confirmed the linearity of both methods, within the studied ranges.

Similarly, Figure 4C shows the calibration curves of BCT in Transcutol using fluorescence spectroscopy, where the correlation coefficient of all replicates was ≥0.9998, showing a constant response factor (ratio intensity/concentration) in the concentration range of 0.078 μg/mL to 1.250 μg/mL (Figure 4D). Table 5 shows the response factors obtained, where no statistical differences (*p* > 0.05) were found between replicates after ANOVA analysis.

#### 3.4.2. Sensitivity

According to the standard deviation of the response and the slope of the calibration curves, the LOQ was 2.61 ± 1.21 μg/mL and the LOD was 0.86 ± 0.40 μg/mL using absorption spectroscopy. In contrast, fluorescence spectroscopy led to values two orders of magnitude lower, with an LOD of 0.01 ± 0.00 and LOQ of 0.02 ± 0.01 μg/mL (mean ± SD). Although the absorption spectroscopy presented a higher LOQ, the method was sensitive enough sensitivity to quantify the samples of the uptake phase in the recovery study and most of the samples in the solubility study. For those samples with a low concentration of drug, such as some solvents in the solubility study and samples from the extraction phase in the recovery study, a more sensitive method was required, which was provided by the fluorescence spectroscopy.

#### 3.4.3. Accuracy and Precision

Accuracy and precision were calculated from the mean of the back-calculated concentrations of calibration curves in both methods, spectrophotometry (Table 7) and fluorimetry (Table 8). Accuracy was expressed as the relative error (%RE) with respect to the nominal concentration, whereas precision was expressed as the relative standard deviation (%RSD) of the predicted concentrations.

For the spectrophotometric method, the relative error obtained was −0.28% ± 1.59%, whereas the relative standard deviation was 2.21% ± 1.93%. Similarly, using the fluorometric method, the relative error was −0.41% ± 1.76%, whereas the relative standard deviation was 1.30% ± 0.67%. In all cases, the values found were much lower than the 15% limit value in accordance with EMA guidelines [41]. Therefore, the method was considered accurate and precise within the concentration range 6.25–60 μg/mL for the spectrophotometric method and within the concentration range 0.078–1.248 μg/mL for the fluorometric method.

In addition to being accurate, precise, linear, and sensitive enough, an analytical method should also be specific for the analyte. Since changes in the matrix of the samples may affect the results obtained, to ensure that the results of the drug uptake and drug recovery were not affected by the tissues, controls were used in the analysis. The controls were pieces of the tissues (sclera, corneal, sublingual, buccal, and skin) incubated in Transcutol (the solvent) without the drug. The tissues underwent the same procedure and conditions as the samples, and, after the incubation and extraction process, the supernatant was analyzed by spectrophotometry or fluorimetry to evaluate any signal in the analysis. The absorbance/intensity of the controls was subtracted from the values obtained in the samples with baricitinib; doing so, any signal that may have come from the tissue was countered, yielding the drug’s concentration within the tissue.

#### 3.4.4. Stability

The stability of the BCT solution in Transcutol was evaluated at two different concentrations, 60 µg/mL (Table 9) and 6.25 µg/mL (Table 10), using the spectrophotometric method and at two different concentrations, 1.25 µg/mL (Table 11) and 0.078 µg/mL (Table 12), using the fluorometric method. The solutions were analyzed freshly prepared and after storage in different conditions: room temperature (22 ± 2 °C), refrigerator (5 ± 3 °C), or freezer (−20 ± 5 °C). The spectrophotometric method was used to analyze the stability of the two highest concentrations, whereas the fluorometric method was used for the two lowest concentrations. The relative difference of the solutions stored at different conditions was below 2% for both methods, leading to the conclusion that BCT is stable in Transcutol for at least 7 days; thus, the samples could be safely stored at 22 °C, 5 °C, or −20 °C for 1 week.

The main limitations of this work are that not all the solvents are suitable as media for the spectrophotometer and fluorimeter. The following solvents were also tested but showed high absorbance values by themselves at the baricitinib wavelength and, thus, were ruled out of the solubility study: polyethylene glycol 400, Tween-80, castor oil, Labrafil, Plurol Oleique, 1-decanol, octanoic acid, and oleic acid.

In other words, although the spectrophotometer and fluorimeter are time-saving and inexpensive techniques with regard to chromatographic methods, some solvents interfere with the analysis and are not suitable.

## 4. Conclusions

As BCT shows low solubility and low permeability, the solubility of this drug was estimated in different solvents, with the purpose of finding those which are suitable for the future development of formulations which permit increasing its availability in tissues. Among the different solvents tested, Transcutol permitted its dissolution at concentrations up to 10.8 mg/mL, and it was selected because of its reported biocompatibility and low toxicity in the literature.

Moreover, the amount of BCT uptake by the different tissues was determined (human skin and porcine mucosae: buccal, sublingual, and vaginal mucosae, cornea, and sclera). The amount of BCT that could be extracted back out of the tissues was also investigated, leading to the percentage of drug recovery for each tissue.

To conduct the sample analysis in this study, two simple, fast, and inexpensive methods were developed and validated for the analysis of BCT in Transcutol, using either absorption spectroscopy (spectrophotometric method) or fluorescence spectroscopy (fluorometric method). Both methods were shown to be linear, precise, and accurate, and they are complimentary to each other, where absorption spectroscopy met these characteristics at higher concentrations, in the range of 6.25 to 60 μg/mL, whereas fluorescence spectroscopy met them at lower concentrations, in the range 0.078 to 1.25 μg/mL. Therefore, both analytical methods are cost and time-effective and are linear, precise and accurate for the analysis of BCT in samples of different tissues. The choice of the method only depends on the concentration of BCT in samples, where the higher sensitivity of fluorescence spectroscopy permits the analysis of much lower concentrations. Solutions of BCT in Transcutol in the whole range of concentrations are stable for up to 7 days at room temperature (22 °C), in the refrigerator (5 °C), and in the freezer (−20 °C), as analyzed by both methods, indicating the suitability of using this solvent for future formulations of BCT.

## Figures and Tables

**Table 1:** Solubility of BCT in aqueous solutions tested by absorption spectroscopy.

Solvent	Solubility (µg/mL)
PBS 7.4: Transcutol (1:1 *v*/*v*)	2033.71 ± 0.74
PBS 7.4: Transcutol (95:5 *v*/*v*)	25.21 ± 0.67
PBS pH 7.4	16.18 ± 0.74
Water	10.86 ± 0.67

PBS: phosphate buffered saline.

**Table 2:** Molecular weight, lipophilia, and estimated solubility of BCT in different oils and enhancers by absorption spectroscopy. Results are reported from the highest to the lower solubility of BCT in the solvents.

Popular Name	Chemical Name (IUPAC)	Molecular Weight (g/mol)	Lipophilia (Log P)	Solubility (µg/mL)
N-Ethyl pyrrolidone	1-Ethylpyrrolidin-2-one	113.16	−0.04	69518 ± 1390
Transcutol	Diethylen glycol monoethyl ether	134.17	−0.54	10817 ± 325
Capryol 90	2-Hydroxypropyl octanoate	202.29		6513 ± 261
Lauroglycol 90	2-Hydroxypropyl dodecanoate	258.4	1.14	600.19 ± 24.01
Labrafac	A mixture of medium-chain triglycerides mainly from caprylic (C8) and capric (C10) acids	NA	NA	503.74 ± 25.19
Surfadone LP-100	1-Octylpyrrolidin-2-one	197.32	NA	93.83 ± 4.69
1-Decanol	Decan-1-ol	158.28	4.57	90.74 ± 5.44
Isostearyl isostearate	16-Methylheptadecyl 16-methylheptadecanoate	537	NA	55.76 ± 3.90
Azone	2-Hydroxypropyl octanoate	202.29	NA	53.52 ± 4.28
Sebacic acid	Decanedioic acid	202.25	2.20	49.24 ± 4.43
Lauryl sulfate	Dodecyl hydrogen sulfate	266.4	NA	23.07 ± 0.31
α-Pinene	2,6,6-Trimethylbicyclo[3.1.1]hept-2-ene	136.23	4.83	3.79 ± 0.09

NA: Not available.

**Table 3:** Solubility of BCT in the oils and enhancers tested using fluorescence spectroscopy. Results are reported from the highest to the lower solubility of BCT in the solvents.

Popular Name	Chemical Name (IUPAC)	Molecular Weight (g/mol)	Lipophilia (Log P)	Solubility (µg/mL)
Paraffin	Paraffinum liquidum	436.84	NA	0.29 ± 0.01
Castor oil	2,3-Bis[[(Z)-12-hydroxyoctadec-9-enoyl]oxy]propyl(Z)-12-hydroxyoctadec-9-enoate	933.4	NA	0.20 ± 0.01
N-Methyl pyrrolidone	1-Methyl-2-pyrrolidinone	99.13	−0.38	0.08 ± 0.00
Limonene 97%	(4R)-1-Methyl-4-prop-1-en-2-ylcyclohexene	136.23	4.57	0.01 ± 0.00

NA: not available.

**Table 4:** Drug uptake (determined by the amount of drug that has penetrated the tissue) and recovery of BCT in the tissues tested. The sample analysis was carried out by spectrophotometry and/or fluorimetry depending on the sample concentration.

	Penetration (µg/g) ^a^	Recovery (µg/g) ^a^	Recovery (%)
Skin	38.79 ± 4.27	1.79 ± 0.19	4.61 ± 4.49
Buccal	208.68 ± 25.04	34.31 ± 4.12	16.44 ± 1.97
Sublingual	20.05 ± 2.60	5.02 ± 0.65	25.01 ± 3.25
Vaginal	61.54 ± 8.62	2.22 ± 0.33	3.61 ± 0.54
Cornea	48.12 ± 7.70	13.24 ± 2.12	27.52 ± 4.40
Sclera	27.03 ± 4.59	2.02 ± 0.34	7.46 ± 1.27

^a^ µg of BCT per g of tissue.

**Table 5:** Linearity of the spectrophotometric method using ANOVA test for the response factor of the calibration curves.

	R1	R2	R3	R4	R5
Conc	Abs/Conc	Abs/Conc	Abs/Conc	Abs/Conc	Abs/Conc
6.25	0.03	0.03	0.03	0.03	0.04
7.50	0.03	0.03	0.03	0.04	0.03
10.00	0.03	0.03	0.03	0.04	0.03
15.00	0.03	0.03	0.03	0.03	0.03
20.00	0.03	0.03	0.03	0.03	0.03
25.00	0.03	0.03	0.03	0.03	0.03
30.00	0.03	0.03	0.03	0.03	0.03
40.00	0.03	0.03	0.03	0.03	0.03
50.00	0.03	0.03	0.03	0.03	0.03
60.00	0.03	0.03	0.03	0.03	0.03

**Table 6:** Linearity of the fluorometric method by ANOVA test of the response factor of the calibration curves.

	R1	R2	R3	R4	R5
Conc	Int/Conc	Int/Conc	Int/Conc	Int/Conc	Int/Conc
0.078	784.3	790.8	795.4	784.3	789.4
0.156	783.1	784.6	797.2	792.6	784.4
0.313	791.0	794.8	797.2	788.2	782.4
0.625	782.8	777.6	777.6	787.4	798.2
1.250	781.0	780.1	785.9	790.1	784.0

**Table 7:** Accuracy and intermediate precision of the spectrophotometric method.

Theoretical Concentration (µg/mL)	Mean Experimental Concentration (µg/mL)	SD (µg/mL)	RE (%)	RSD (%)
6.25	6.276	0.449	−0.42	7.16
7.5	7.304	0.190	2.61	2.61
10	9.770	0.299	2.30	3.06
15	14.740	0.301	1.73	2.04
20	20.220	0.319	−1.10	1.58
25	25.604	0.285	−2.41	1.11
30	30.219	0.665	−0.73	2.20
40	39.715	0.344	0.71	0.87
50	50.123	0.540	−0.25	1.08
60	59.778	0.208	0.37	0.35
Mean (%)	-	-	−0.28	2.21
SD	-	-	1.59	1.93

SD: standard deviation; RE: relative error; RSD: relative standard deviation.

**Table 8:** Accuracy and intermediate precision of the fluorometric method.

Theoretical Concentration (µg/mL)	Mean (µg/mL)	SD (µg/mL)	RE (%)	RSD (%)
0.078	0.075	0.001	−3.34	1.97
0.156	0.156	0.002	0.31	1.38
0.312	0.316	0.005	1.34	1.67
0.624	0.622	0.008	−0.36	1.28
1.248	1.248	0.003	0.01	0.21
Mean (%)	-	-	−0.41	1.30
SD	-	-	1.76	0.67

SD: standard deviation; RE: relative error; RSD: relative standard deviation.

**Table 9:** Stability of the standard solution 60 µg/mL stored at three different temperatures: room temperature, fridge, and freezer, as analyzed by absorption spectroscopy (spectrophotometry).

	Room Temperature		Fridge		Freezer	
Stability Time (Day)	Absorbance (AU)	Relative Difference (%)	Absorbance (AU)	Relative Difference (%)	Absorbance (AU)	Relative Difference (%)
0 day	1.7628	N/A	1.7643	N/A	1.7535	N/A
1 day	1.7222	−2.30%	1.7608	−0.20%	1.7545	0.06%
7 days	1.7325	−1.72%	1.7297	−1.96%	1.7521	−0.08%

**Table 10:** Stability of the standard solution of 6.25 µg/mL stored at different temperatures: room temperature, fridge, and freezer, as analyzed by absorption spectroscopy (spectrophotometry).

	Room Temperature		Fridge		Freezer	
Stability Time (Day)	Absorbance (AU)	Relative Difference (%)	Absorbance (AU)	Relative Difference (%)	Absorbance (AU)	Relative Difference (%)
0 day	0.1899	N/A	0.1969	N/A	0.227	0.23
1 day	0.1897	−0.11%	0.191	−3.00%	1.7545	0.57%
7 days	0.1871	−1.47%	0.1985	0.81%	1.7521	−0.88 %

**Table 11:** Stability of the standard solution 1.25 µg/mL stored at the different temperatures: room temperature, fridge, and freezer, as analyzed by fluorescence spectroscopy (fluorimetry).

	Room Temperature		Fridge		Freezer	
Stability Time (Day)	Intensity (AU)	Relative Difference (%)	Intensity (AU)	Relative Difference (%)	Intensity (AU)	Relative Difference (%)
0 day	975.23	N/A	982.4	N/A	982.44	N/A
1 day	974.26	−0.10%	971.62	−1.10%	976.25	−0.63%
7 days	975.16	−0.01%	982.42	0.00%	976.25	−0.63%

**Table 12:** Stability of the standard solution 0.078 µg/mL stored at three different temperatures: room temperature, fridge, and freezer, as analyzed by fluorescence spectroscopy (fluorimetry).

	Room Temperature		Fridge		Freezer	
Stability Time (day)	Intensity (AU)	Relative Difference (%)	Intensity (AU)	Relative Difference (%)	Intensity (AU)	Relative Difference (%)
0 day	61.27	N/A	62.24	N/A	61.24	N/A
1 day	61.18	−0.15%	61.27	−1.56%	61.24	0.00%
7 days	61.79	0.85%	62.03	−0.34%	61.25	0.02%

## Data Availability

The data presented in this study are available on request from the corresponding author. The data are not publicly available due to them being part of a PhD thesis; they will be made available once the thesis has been published.

## References

[1] Alexander A., Dwivedi S., Ajazuddin, Giri T.K., Saraf S., Saraf S., Tripathi D.K. (2012). Approaches for Breaking the Barriers of Drug Permeation through Transdermal Drug Delivery. J. Control. Release.

[2] Lam J.K.W., Cheung C.C.K., Chow M.Y.T., Harrop E., Lapwood S., Barclay S.I.G., Wong I.C.K. (2020). Transmucosal Drug Administration as an Alternative Route in Palliative and End-of-Life Care during the COVID-19 Pandemic. Adv. Drug. Deliv. Rev..

[3] Drug Bank Baricitinib: Uses, Interactions, Mechanism of Action. https://go.drugbank.com/drugs/DB11817.

[4] National Center for Biotechnology Information PubChem Database. PubChem Compound Summary for CID 44205240, Baricitinib. https://pubchem.ncbi.nlm.nih.gov/compound/Baricitinib.

[5] Nezamololama N., Fieldhouse K., Metzger K., Gooderham M. (2020). Emerging Systemic JAK Inhibitors in the Treatment of Atopic Dermatitis: A Review of Abrocitinib, Baricitinib, and Upadacitinib. Drugs Context.

[6] Nakashima C., Yanagihara S., Otsuka A. (2022). Innovation in the Treatment of Atopic Dermatitis: Emerging Topical and Oral Janus Kinase Inhibitors. Allergol. Int..

[7] (2014). EMA Ficha Técnica Baricitinib.

[8] U.S. FDA (2022). Prescribing Information OLUMIANT (Baricitinib) Tablets.

[9] Marconi V.C., Ramanan A.V., de Bono S., Kartman C.E., Krishnan V., Liao R., Piruzeli M.L.B., Goldman J.D., Alatorre-Alexander J., de Cassia Pellegrini R. (2021). Efficacy and Safety of Baricitinib for the Treatment of Hospitalised Adults with COVID-19 (COV-BARRIER): A Randomised, Double-Blind, Parallel-Group, Placebo-Controlled Phase 3 Trial. Lancet Respir. Med..

[10] Shi J.G., Chen X., Lee F., Emm T., Scherle P.A., Lo Y., Punwani N., Williams W.V., Yeleswaram S. (2014). The Pharmacokinetics, Pharmacodynamics, and Safety of Baricitinib, an Oral JAK 1/2 Inhibitor, in Healthy Volunteers. J. Clin. Pharmacol..

[11] Reich K., Kabashima K., Peris K., Silverberg J.I., Eichenfield L.F., Bieber T., Kaszuba A., Kolodsick J., Yang F.E., Gamalo M. (2020). Efficacy and Safety of Baricitinib Combined with Topical Corticosteroids for Treatment of Moderate to Severe Atopic Dermatitis: A Randomized Clinical Trial. JAMA Dermatol..

[12] Alshetaili A.S. (2019). Solubility and Solution Thermodynamics of Baricitinib in Six Different Pharmaceutically Used Solvents at Different Temperatures. Z. Phys. Chem..

[13] Markham A. (2017). Baricitinib: First Global Approval. Drugs.

[14] Zheng X.Q., Huang J.F., Lin J.L., Zhu Y.X., Wang M.Q., Guo M.L., Zan X.J., Wu A.M. (2021). Controlled Release of Baricitinib from a Thermos-Responsive Hydrogel System Inhibits Inflammation by Suppressing JAK2/STAT3 Pathway in Acute Spinal Cord Injury. Colloids Surf. B Biointerfaces.

[15] Hiraganahalli Bhaskarmurthy D., Evan Prince S. (2021). Effect of Baricitinib on TPA-Induced Psoriasis like Skin Inflammation. Life Sci..

[16] Anwer M.K., Ali E.A., Iqbal M., Ahmed M.M., Aldawsari M.F., Al Saqr A., Ansari M.N., Aboudzadeh M.A. (2022). Development of Sustained Release Baricitinib Loaded Lipid-Polymer Hybrid Nanoparticles with Improved Oral Bioavailability. Molecules.

[17] Ansari M.J., Alshahrani S.M. (2019). Nano-Encapsulation and Characterization of Baricitinib Using Poly-Lactic-Glycolic Acid Co-Polymer. Saudi Pharm. J..

[18] Ma T.Y., Hollander D., Krugliak P., Katz K. (1990). PEG 400, a Hydrophilic Molecular Probe for Measuring Intestinal Permeability. Gastroenterology.

[19] Thakur S., Bajwa N., Baldi A. (2018). New analytical methods for estimation of arteether by uv and fluorescence spectrophotometry: Development and validation. J. Drug Deliv. Ther..

[20] Karimi M., Mashreghi M., Saremi S.S., Jaafari M.R. (2020). Spectrofluorometric Method Development and Validation for the Determination of Curcumin in Nanoliposomes and Plasma. J. Fluoresc..

[21] Fraihat S.M., al Khatib H.S. (2020). Development and Validation of Spectrophotometric and Spectrofluorimetric Methods for Determination of Cilnidipine. Trop. J. Pharm. Res..

[22] Gandhi S.V., Kapoor B.G. (2019). Development and Validation of UV Spectroscopic Method for Estimation of Baricitinib. J. Drug Deliv. Ther..

[23] Derayea S.M., Omar M.A., Abdel-Lateef M.A.K., Hassan A.I. (2016). Development and Validation of a New Spectrofluorimetric Method for the Determination of Some Beta-Blockers through Fluorescence Quenching of Eosin Y. Application to Content Uniformity Test. Open Chem..

[24] Hadgraft J. (1999). Passive Enhancement Strategies in Topical and Transdermal Drug Delivery. Int. J. Pharm..

[25] National Center for Biotechnology Information PubChem Database. Diethylene Glycol Monoethyl Ether | C6H14O3—PubChem. https://pubchem.ncbi.nlm.nih.gov/compound/Diethylene-glycol-monoethyl-ether.

[26] Sullivan D.W., Gad S.C., Julien M. (2014). A Review of the Nonclinical Safety of Transcutol^®^, a Highly Purified Form of Diethylene Glycol Monoethyl Ether (DEGEE) Used as a Pharmaceutical Excipient. Food Chem. Toxicol..

[27] Pérez-González N., De Febrer N.B., Calpena-Campmany A.C., Nardi-Ricart A., Rodríguez-Lagunas M.J., Morales-Molina J.A., Soriano-Ruiz J.L., Fernández-Campos F., Clares-Naveros B. (2021). New Formulations Loading Caspofungin for Topical Therapy of Vulvovaginal Candidiasis. Gels.

[28] Predic Atkinson J., Maibach H.I., Dragicevic N., Nina D., Maibach H.I. (2015). Targets in Dermal and Transdermal Delivery and Classification of Penetration Enhancement Methods. Percutaneous Penetration Enhancers Chemical Methods in Penetration Enhancement: Drug Manipulation Strategies and Vehicle Effects.

[29] Mallandrich M., Fernández-Campos F., Clares B., Halbaut L., Alonso C., Coderch L., Garduño-Ramírez M.L., Andrade B., del Pozo A., Lane M.E. (2017). Developing Transdermal Applications of Ketorolac Tromethamine Entrapped in Stimuli Sensitive Block Copolymer Hydrogels. Pharm. Res..

[30] Moussaoui S.E., Fernández-Campos F., Alonso C., Limón D., Mallandrich M. (2021). Topical Mucoadhesive Alginate-Based Hydrogel Loading Ketorolac for Pain Management after Pharmacotherapy, Ablation, or Surgical Removal in Condyloma Acuminata. Gels.

[31] Araújo J., Garcia M.L., Mallandrich M., Souto E.B., Calpena A.C. (2012). Release Profile and Transscleral Permeation of Triamcinolone Acetonide Loaded Nanostructured Lipid Carriers (TA-NLC): In Vitro and Ex Vivo Studies. Nanomedicine.

[32] Mallandrich M., Calpena A.C., Clares B., Parra A., Garcia M.L., Soriano J.L., Fernandez-Campos F. (2021). Nano-Engineering of Ketorolac Tromethamine Platforms for Ocular Treatment of Inflammatory Disorders. Nanomedicine.

[33] Silva-Abreu M., Espinoza L.C., Rodríguez-Lagunas M.J., Fábrega M.J., Espina M., García M.L., Calpena A.C. (2017). Human Skin Permeation Studies with PPARγ Agonist to Improve Its Permeability and Efficacy in Inflammatory Processes. Int. J. Mol. Sci..

[34] Sanz R., Clares B., Mallandrich M., Suñer-Carbó J., Montes M.J., Calpena A.C. (2018). Development of a Mucoadhesive Delivery System for Control Release of Doxepin with Application in Vaginal Pain Relief Associated with Gynecological Surgery. Int. J. Pharm..

[35] Amores S., Domenech J., Colom H., Calpena A.C., Clares B., Gimeno Á., Lauroba J. (2014). An Improved Cryopreservation Method for Porcine Buccal Mucosa in Ex Vivo Drug Permeation Studies Using Franz Diffusion Cells. Eur. J. Pharm. Sci..

[36] Gimeno A., Calpena A.C., Sanz R., Mallandrich M., Peraire C., Clares B. (2014). Transbuccal Delivery of Doxepin: Studies on Permeation and Histological Investigation. Int. J. Pharm..

[37] Parra A., Mallandrich M., Clares B., Egea M.A., Espina M., García M.L., Calpena A.C. (2015). Design and Elaboration of Freeze-Dried PLGA Nanoparticles for the Transcorneal Permeation of Carprofen: Ocular Anti-Inflammatory Applications. Colloids Surf. B Biointerfaces.

[38] Limón D., Jiménez-Newman C., Calpena A.C., González-Campo A., Amabilino D.B., Pérez-García L. (2017). Microscale Coiling in Bis-Imidazolium Supramolecular Hydrogel Fibres Induced by Release of a Cationic Serine Protease Inhibitor (ESI). Chem. Commun..

[39] Limón D., Jiménez-Newman C., Rodrigues M., González-Campo A., Amabilino D.B., Calpena A.C., Pérez-García L. (2017). Cationic Supramolecular Hydrogels for Overcoming the Skin Barrier in Drug Delivery. ChemistryOpen.

[40] Limón D., Talló Domínguez K., Garduño-Ramírez M.L., Andrade B., Calpena A.C., Pérez-García L. (2019). Nanostructured Supramolecular Hydrogels: Towards the Topical Treatment of Psoriasis and Other Skin Diseases. Colloids Surf. B Biointerfaces.

[41] Committee for Medicinal Products for Human Use (CHMP) (2011). European Medicines Agency Guideline on Bioanalytical Method Validation.

[42] Asociación Española de Farmacéuticos de la Industria (2001). Validación de Métodos Analíticos.

[43] Kim J.Y., Kim S., Papp M., Park K., Pinal R. (2010). Hydrotropic Solubilization of Poorly Water-Soluble Drugs. J. Pharm. Sci..

[44] USP 38 (2015). General Notices and Requirements. Description and Solubility.

[45] Savjani K.T., Gajjar A.K., Savjani J.K. (2012). Drug Solubility: Importance and Enhancement Techniques. ISRN Pharm..

[46] Gupta S., Kesarla R., Omri A. (2013). Formulation Strategies to Improve the Bioavailability of Poorly Absorbed Drugs with Special Emphasis on Self-Emulsifying Systems. ISRN Pharm..

[47] Miyako Y., Khalef N., Matsuzaki K., Pinal R. (2010). Solubility Enhancement of Hydrophobic Compounds by Cosolvents: Role of Solute Hydrophobicity on the Solubilization Effect. Int. J. Pharm..

[48] Sareen S., Joseph L., Mathew G. (2012). Improvement in Solubility of Poor Water-Soluble Drugs by Solid Dispersion. Int. J. Pharm. Investig..

[49] EMA (2016). Assessment Report Olumiant.

[50] Australian Government (2019). Australian Public Assessment Report for Baricitinib.

[51] Kumar Bolla P., Clark B.A., Juluri A., Cheruvu H.S., Renukuntla J. (2020). Pharmaceutics Evaluation of Formulation Parameters on Permeation of Ibuprofen from Topical Formulations Using Strat-M^®^ Membrane. Pharmaceutics.

[52] Gad S.C., Cassidy C.D., Aubert N., Spainhour B., Robbe H. (2006). Nonclinical Vehicle Use in Studies by Multiple Routes in Multiple Species. Int. J. Toxicol..

[53] Silva-Abreu M., Espinoza L.C., Halbaut L., Espina M., García M.L., Calpena A.C. (2018). Comparative Study of Ex Vivo Transmucosal Permeation of Pioglitazone Nanoparticles for the Treatment of Alzheimer’s Disease. Polymers.

[54] Gómez-Segura L., Parra A., Calpena A.C., Gimeno Á., Boix-Montañes A. (2020). Carprofen Permeation Test through Porcine Ex Vivo Mucous Membranes and Ophthalmic Tissues for Tolerability Assessments: Validation and Histological Study. Vet. Sci..

[55] Miralles-Cardiel E., Silva-Abreu M., Calpena A.C., Casals I. (2021). Development and Validation of an Hplc–Ms/Ms Method for Pioglitazone from Nanocarriers Quantitation in Ex Vivo and in Vivo Ocular Tissues. Pharmaceutics.

[56] Gartner L.P., Gartner L.P. (2021). Integument. Textbook of Histology.

[57] Kuo S.-H., Shen C.-J., Shen C.-F., Cheng C.-M. (2020). Role of PH Value in Clinically Relevant Diagnosis. Diagnostics.

[58] El-Hammadi M.M., Arias J.L. (2021). Nanomedicine for Vaginal Drug Delivery. Theory Appl. Nonparenter. Nanomed..

[59] Rohan L.C., Sassi A.B. (2009). Vaginal Drug Delivery Systems for HIV Prevention. Am. Assoc. Pharm. Sci..

[60] Gökmen O., Yeşilırmak N., Akman A., Güngör S.G., Yücel A.E., Yeşil H., Yıldız F., Sise S., Diakonis V. (2017). Corneal, Scleral, Choroidal, and Foveal Thickness in Patients with Rheumatoid Arthritis. Turk. J. Ophthalmol..

[61] Aungst B., Svensson C.K., Gaudana R., Ananthula H.K., Parenky A., Mitra A.K. (2010). Ocular Drug Delivery. Am. Assoc. Pharm. Sci..

[62] Leal J., Smyth H.D.C., Ghosh D. (2017). Physicochemical Properties of Mucus and Their Impact on Transmucosal Drug Delivery. Int. J. Pharm..

[63] Boegh M., Nielsen H.M. (2014). Mucus as a Barrier to Drug Delivery-Understanding and Mimicking the Barrier Properties. Basic Clin. Pharmacol. Toxicol..

[64] Choi S.A., Park E.J., Lee J.H., Min K.A., Kim S.T., Jang D.-J., Maeng H.-J., Jin S.G., Cho K.H. (2022). Preparation and Characterization of Pazopanib Hydrochloride-Loaded Four-Component Self-Nanoemulsifying Drug Delivery Systems Preconcentrate for Enhanced Solubility and Dissolution. Pharmaceutics.

[65] Date A.A., Nagarsenker M.S. (2019). Single-Step and Low-Energy Method to Prepare Solid Lipid Nanoparticles and Nanostructured Lipid Carriers Using Biocompatible Solvents. Eur. J. Pharm. Res..

[66] Lee S.G., Kang J.B., Kim S.R., Kim C.J., Yeom D.W., Yoon H.Y., Kwak S.S., Choi Y.W. (2016). Enhanced Topical Delivery of Tacrolimus by a Carbomer Hydrogel Formulation with Transcutol P. Drug Dev. Ind. Pharm..

